# Optimization of solid lipid nanoparticles prepared by a single emulsification-solvent evaporation method

**DOI:** 10.1016/j.dib.2015.11.038

**Published:** 2015-11-25

**Authors:** Deep Pooja, Lakshmi Tunki, Hitesh Kulhari, Bharathi B. Reddy, Ramakrishna Sistla

**Affiliations:** a Medicinal Chemistry & Pharmacology Division, CSIR-Indian Institute of Chemical Technology, Hyderabad 500007, India; b IICT-RMIT Joint Research Centre, CSIR-Indian Institute of Chemical Technology, Hyderabad, India; c Health Innovations Research Institute, RMIT University, Melbourne, Australia

**Keywords:** Solid lipid nanoparticles, Single emulsification-solvent evaporation, Optimization, Formulation parameters, Process variables

## Abstract

This data article contains the data related to the research article “Characterization, biorecognitive activity and stability of WGA grafted lipid nanostructures for the controlled delivery of rifampicin” (Pooja et al. 2015) [1]. In the present study, SLN were prepared by a single emulsification-solvent evaporation method and the various steps of SLN preparation are shown in a flow chart. The preparation of SLN was optimized for various formulation variables including type and quantity of lipid, surfactant, amount of co-surfactant and volume of organic phase. Similarly, effect of variables related to homogezation, sonication and stirring processes, on the size and surface potential of SLN was determined and optimized.

**Specifications Table**

**Table:** 

Subject area	Chemistry, lipids and biology
More specific subject area	Targeted nanomedicine
Type of data	Table and figure
How data was acquired	Particle size, polydispersity index and surface charge (Zetasizer, NanoZS, Malvern)
Data format	Raw and analyzed
Experimental factors	Formulation and process parameters were changed for optimization of size and zeta potential of nanoparticles.
Experimental features	Various formulations were prepared by single emulsification- solvent evaporation method to get nanoparticles of desired size and zeta potential.
Data source location	NA
Data accessibility	The data are presented in this article

**Value of data**

• The article describes the preparation, optimization and characterization of solid lipid nanoparticles.
• The data can be useful for other researchers investigating the effects of different lipids and surfactants on size and surface charge of nanoparticles.
• The optimized formulation parameters could be used for the development of solid lipid nanoparticles of hydrophobic drugs.

## 1 Experimental design, material and methods

**Fig. 1:**
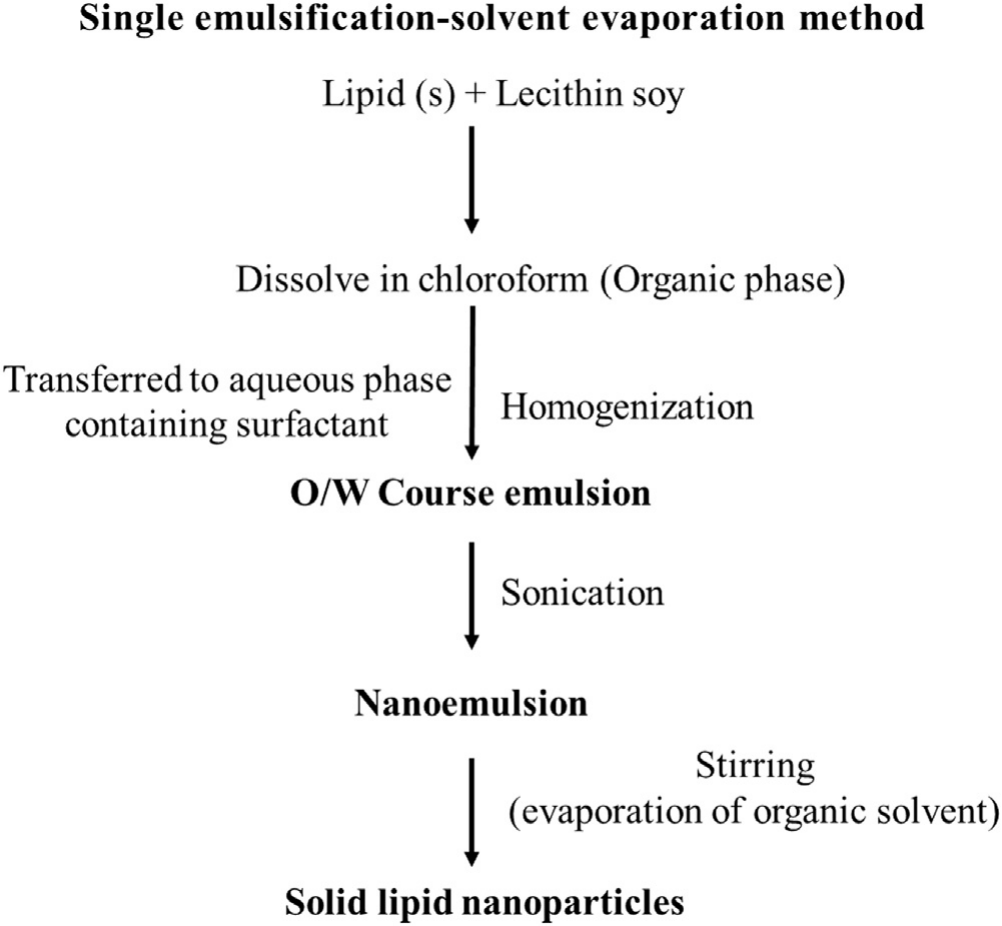
Flow chart representing the preparation of solid lipid nanoparticles.

Solid lipid nanoparticles (SLN) i.e. lipid nanoparticles with solid matrix is the most fascinating carrier for oral drug delivery because of their excellent biocompatibility, high drug loading, long-term stability and feasibility for large scale production [1], [2], [3], [4], [5]. In this study, solid lipid nanoparticles (SLN) were prepared by a single emulsification-solvent evaporation method. Fig. 1 presents the various steps of preparation of SLN. Various formulation parameters (Table 1) and process variables (Table 2) were optimized on the basis of their effect on particle size, polydispersity index and zeta potential. These parameters included type and quantity of lipid and surfactant, quantity of co-surfactant, volume of organic phase, homogenization speed and time, sonication time, stirring speed and time. Formulations were prepared by changing one parameter at a time while keeping other parameters constant.

### 1.1 Optimization of formulation variables

#### 1.1.1 Type and quantity of lipids

Three different lipids viz. glyceryl monostearte (GMS), tristearin and tripalmitin were used as lipid matrix. The particle diameter (PD), polydispersity index (PDI) and zeta potential (ZP) were measured using a Zetasizer NanoZS (Malvern, UK). The lipid showing minimum PD and PDI was selected and used in three different quantities (80, 100 and 120 mg).

#### 1.1.2 Type and concentration of surfactants

The type and concentration of surfactant affect the particle size as well as stability of nanoparticles. At low concentration, surfactant will not be sufficient to cover the surface of nanoparticles resulting into increased particle size due to particle aggregation. High concentration of surfactant may lead to bridging between nanoparticles and may also cause toxicity. Therefore, three different surfactants (Tween®80, Poloxomer 188 and polyvinyl alcohol) were evaluated at three different concentrations (1%, 1.5% and 2% w/v).

#### 1.1.3 Volume of organic phase

The organic solvent is used to dissolve the lipids and chloroform was used in this study in varying volumes (1–5 mL). The formulation showing good particle size with minimum volume of solvent was selected.

#### 1.1.4 Quantity of co-surfactant

Lecithin soy was used as co-surfactant which act as internal emulsifier and favors to particle size reduction and stability. Lecithin soy was used at different concentration (20, 30 and 40) to get a formulation having small particle size, less PDI with good zeta potential and stability.

### 1.2 Optimization of process variables

#### 1.2.1 Homogenization speed and time, sonication time and stirring speed and time

The organic phase was poured in aqueous surfactant phase and homogenized at different speed (5000, 8000 and 11000 rpm) for different time (3, 4, 5 and 6 min) to get course emulsion. Then this course emulsion was sonicated for different time period to get a nanoemulsion. Finally formulation was stirred to evaporate the organic solvent and to get the nanoparticles. The formulation was stirred at different speed (800, 1000, and 1200 rpm) and for different time period (1, 2 and 3 h) for optimization.

## Figures and Tables

**Table 1:** Optimization of various formulation parameters for the preparation of solid lipid nanoparticles.

Formulation	Variable		PD (nm)	PDI	ZP (mV)
*Type of lipid*					
F1	GMS	100	55.53±2.4	0.23±0.04	−23.2±2.1
F2	Tristearin	100	157.5±6.7	0.35±0.11	−26.9±2.3
F3	Tripalmitin	100	119.5±3.9	0.43±0.08	−22.1±1.9
*Quantity of lipid* (mg)					
F4	GMS	80	49.28±3.1	0.27±0.09	−21.8±1.6
F1	GMS	100	55.53±2.4	0.23±0.04	−23.2±2.1
F6	GMS	120	55.09±3.7	0.30±0.02	−29.7±2.3
*Type and concentration of surfactant* (%w/v)					
F7	Tween 80	1	66.67±2.5	0.36±0.12	−31.8±2.4
F1	Tween 80	1.5	55.53±2.4	0.23±0.04	−23.2±2.1
F8	Tween 80	2	133.2±5.6	0.27±0.08	−26.6±1.8
F9	Poloxomer 188	1	61.4±4.4	0.38±0.09	−29.9±2.3
F10	Poloxomer 188	1.5	65.7±3.9	0.40±0.12	−26.4±2.1
F11	Poloxomer 188	2	64.9±2.8	0.39±0.10	−23.5±2.5
F12	PVA	1	120.92±6.1	0.15±0.09	−32.0±1.9
F13	PVA	1.5	108.84±4.3	0.20±0.07	−26.6±2.4
F14	PVA	2	102.86±4.8	0.21±0.11	−24.5±1.8
*Volume of organic solvent* (mL)					
F15	CHCl_3_	1	48.91±2.4	0.36±0.11	−19.6±1.4
F16	CHCl_3_	2	52.81±1.9	0.21±0.07	−24.3±2.6
F1	CHCl_3_	3	55.53±2.4	0.23±0.04	−23.2±2.1
F17	CHCl_3_	5	47.73±2.6	0.25±0.04	−23.7±2.3
*Quantity of co-surfactant (mg)*					
F16	lecithin soy	20	52.81±1.9	0.21±0.07	−24.3±2.6
F18	lecithin soy	30	47.54±2.3	0.21±0.09	−25.5±1.8
F19	lecithin soy	40	50.32±3.1	0.28±0.10	−28.6±2.4

GMS: Glyceryl monostearte; PVA: Polyvinyl alcohol; PD: Particle diameter, PDI: Polydispersity index; ZP: Zeta potential.

**Table 2:** Optimization of various process variables for preparation solid lipid nanoparticles.

Formulation	Variable	PD (nm)	PDI	ZP (mV)
*Homogenization speed* (rpm)				
F20	5000	64.67±4.8	0.56±0.03	−27.5±2.5
F18	8000	47.54±2.3	0.21±0.09	−25.5±1.8
21	11000	44.43±3.1	0.26±0.03	−26.5±2.1
*Homogenization time* (min)				
F22	3	157.92±5.7	0.45±0.05	−30.3±3.1
F23	4	76.21±3.9	0.28±0.07	−25.8±2.8
F21	5	44.43±3.1	0.26±0.03	−26.5±2.1
F24	6	71.23±4.8	0.29±0.11	−23.9±2.7
*Sonication time* (min)				
F25	5	>500	–	–
F26	10	135.45±6.7	0.32±0.13	−27.1±2.9
F21	15	44.43±3.1	0.26±0.03	−26.5±2.1
F27	20	49.89±2.8	0.24±0.09	−25.8±2.6
*Stirring speed* (rpm)				
F28	800	59.02±3.9	0.25±0.05	−20.1±1.9
F21	1000	44.43±3.1	0.26±0.03	−26.5±2.1
F29	1200	67.82±4.2	0.27±0.02	−22.9±2.5
*Stirring time* (h)				
F30	1	69.48±4.5	0.42±0.07	−28.4±2.7
F31	2	57.37±5.1	0.31±0.05	−26.4±1.8
F21	3	44.43±3.1	0.26±0.03	−26.5±2.1
F32	4	61.34±3.8	0.25±0.09	−26.2±2.5

## References

[1] Pooja D., Tunki L., Kulhari H., Reddy B.B., Sistla R. (2015). Characterization, biorecognitive activity and stability of WGA grafted lipid nanostructures for the controlled delivery of rifampicin. Chem. Phys. Lipids.

[2] Dingler A., Gohla S. (2002). Production of solid lipid nanoparticles (SLN): scaling up feasibilities. J. Microencapsul..

[3] Chauhan H., Mohapatra S., Munt D.J., Chandratre S., Dash A. (2015). Physical–chemical characterization and formulation considerations for solid lipid nanoparticles. AAPS PharmSciTech.

[4] Pooja D., Kulhari H., Tunki L., Chinde S., Kuncha M., Grover P., Rachamalla S.S., Sistla R. (2015). Nanomedicines for targeted delivery of etoposide to non-small cell lung cancer using transferrin functionalized nanoparticles. RSC Adv..

[5] Lee M.K., Lim S.J., Kim C.K. (2007). Preparation, characterization and in vitro cytotoxicity of paclitaxel-loaded sterically stabilized solid lipid nanoparticles. Biomaterials.

